# From Our Correspondents

**Published:** 1987-08

**Authors:** 


### From Our Correspondents

### Morals and the Public Health

The hospital in which I spent one arm of my rotating Lecturer/Senior Registrar post is situated close to one of London's several 'red-light' districts. As a result we always had several 'professionals' and as many amateurs occupying beds in our gynae wards because of acute or chronic pelvic inflammatory disease. The weekly Professorial ward round always ended at a four-bed ward which, on the day of the events I wish to relate, was occupied by four young women with acute or chronic PID. They had graphically described their signs and symptoms to the students who relayed them to the Professor and his team in textbook clinical terms. The symptom common to each of them and in all aspects of their life was PAIN. One had described her life as 'living hell' and the others did not disagree.

We adjourned to the seminar room to discuss their problems. My Professor stated that he saw as his main objective to treat these girls "so that they could get back to having a good time". Emboldened by the fact that I was due to move on in the near future, I said, "Excuse me, sir, but could you please tell me what you mean by 'having a good time'?" The reply was not particularly enlightening, "Well you know (nudge, nudge, wink, wink) – having a good time."

I became even bolder. "I presume, sir, that you mean going back to having sexual intercourse with one (or more) of their several partners. I find it difficult to understand how that can be construed as 'having a good time'. They have described their symptoms to us: one has clearly stated that her life is a living hell. How can the state they find themselves in be thought of as 'having a good time'?" By now I was waxing eloquent, but my chief stopped me, "Gordon, you are moralising again and we must not be judgemental." I don't think the word "Poppycock" crossed my lips but it nearly did. "Sir", I continued, very respectfully, "if we had as many patients admitted to this hospital as a result of, say, asbestosis because of a local asbestos factory as we do from PID, the Public Health Department would have closed the factory down years ago. What these girls are suffering from is directly and solely due to their way of life. Unless and until they are advised to change it and act on that advice, they will continue to live in the hell on earth made for them by themselves and their selfish partners." The ward round ended there.

This happened almost 15 years ago but I cannot get it out of my mind now that the plague of AIDS is upon us. I am still told, "You are moralising, Gordon", as though that was unforgivable in this enlightened age. Of course I am, and I make no apology for it. Morals have to do with how we live in relation to our fellow creatures and, for me unequivocably, our Creator. The more we stray from the ethical codes from which our moral actions spring, the more at dis-ease we are within society, and the more disease there is likely to be in that society.

I have never forgotten the following quotation from Time magazine in 1975 quoting a psychoanalyst, "This is a society that is increasingly denying its impotence by calling it tolerance, preaching resignation and naming all this progress."

I am writing this in the middle of 'AIDS week' and want to shout as loudly as I possibly can, "Morals have everything to do with the public health." It is not possible to live in this world (or practise as a doctor for that matter) without taking moral stances or making moral judgements. Abrogating that responsibility is a moral stance in itself. I just cannot understand why this is not glaringly obvious to everyone.

In the specific context of the prevention of AIDS I am drawn inexorably to the following conclusions based on my scientific and medical training:

1. The principle of safer sex using a condom with fewer partners is a partial but fundamentally flawed solution;
2. The only effective action needs to be based on the Judaeo-Christian principles (although not confined to these religions) of no pre-marital sexual intercourse and life-long fidelity to one partner within the marriage bond.

A society which cannot accept this is confusing repression with self-control and licence with liberty.

It is, of course, axiomatic that we must have the greatest compassion for and, as doctors or nurses, a clear responsibility to care for those afflicted by this or any other plague. Those not yet affected also have a clear and unambiguous responsibility to themselves and those around them to take the most effective action possible to prevent it occurring to them. 'Safer sex' as portrayed by the media is akin to closing one gate at a railway level crossing. It suggests you are half expecting a train – ineffective and highly dangerous.

A. G. Stirratt

### The Government's anti-AIDS campaign

We all watch the Television Health Warnings and the Posters as they come upon us with grim discomfort, and no doubt they are making their impact. I suppose it is impossible to strike exactly the right note. The Iceberg conveys the message of hidden menace and is suitably chilling. A poster with a picture of a hetero-sexual couple in bed, with the caption 'Bang, bang, you're dead' seems rather frivolous and wrongly targetted, I suspect Edwina Curry may have chosen that one. Most interesting was the TV documentatry aimed presumably at the audience who would be turned off if anybody looking like an establishment figure was speaking to them. A male actor dressed in a nondescript grey, reminiscent of prisons, looking rather unhealthy and with a non-specific regional accent was paired with a rather smarter looking actress. In words of one syllable they spelt out the horrible facts aided by plaster models. The first model was a female pelvis in sagittal section displaying a rectum and a vagina; with apparent impartiality the use of both these cavities for the purposes of intercourse was described – it didn't matter which it seemed, so long as you used a condom. The second plaster pelvis was male and carried an erect penis. It was ceremonially clothed with a condom by the man who gave the impression that his preference was for the rectum. All very neutral. This presentation conveyed two tacit assumptions, one that anal intercourse is an acceptable alternative activity and the other that heterosexual intercourse plays an equal role in spreading this disease. The former suggestion would be repudiated by the majority and the latter is manifestly untrue.

In San Francisco over a quarter of the adult male population are homosexual, this was the first city in the Western world where the AIDS virus took off from Africa, and in this city at one count, of 25,000 HIV positive individual only 3 were hetero-sexuals. The homo-sexual condition may not be of the individual's own choosing, though some are bi-sexual and many live discretely with a single partner, in effect happily married. These are not the people who have provided the hotbed for AIDS and it will be sad if such lives are made miserable by an anti-homosexual hate campaign. AIDS however has taken off in San Francisco because the promiscuity of most homosexuals there beggars belief. 'Gay' clubs abound and it is from places like these that the infection spreads. This country has it's 'gay' clubs, some of them sponsored by loony left councils, there is even, I was astonished to learn from the minutes of the 1986 BMA Annual Representative Meeting a 'Gay' Medical Association. Such groups should be the subject of close scrutiny by DHSS inspectors, perhaps they are.

Another AIDS programme which I happened to view was on the subject of testing for sero-positivity. In a hypothetical interview a young woman was seeking advice from an AIDS counseller. She feared that she might have contracted the disease. She was advised not to be AIDS tested because it was pointed out to her that nothing could be done if she was found to be infected; if she conducted her future relations in such a way as not to contract the disease she would also not transmit it if she had it. If she was found to be positive she might not be able to get a mortgage or an insurance policy and she would be penalised to no purpose. Knowledge that she had the disease would destroy her future peace of mind – so why be tested?

The Medical 'establishment' view now seems to be emerging. It is that chastity and the condom are the only defences that can be countenanced, voluntary AIDS testing is discouraged except for blood donors, compulsory AIDS testing would be repugnant, isolation of carriers unthinkable. It takes a determined man to press for the unpopular measures in the present moral climate, the erstwhile abhorrence of the homosexual has been replaced by an amused tolerance and the scorn of the sophisticated is directed against the likes of Mary Whitehouse. While this attitude prevails there seems little doubt that AIDS will continue to spread remorselessly unless or until a cure or a vaccine are found. However attitudes might change, to paraphrase Dr Johnson – when a man learns he may be fatally infected at any time it could 'concentrate his mind wonderfully'.

M. G. Wilson

**The Sasakawa Conference Centre at Berkeley**

At a recent meeting of the Jenner trust it was suggested that the Sasakawa Conference Centre would make a good venue for meetings of the various regional and local clinical clubs e.g. West Country Physicians, Surgeons etc. The conversion of Jenner's home at Berkeley into a museum and a conference centre was made possible by the donation of half a million pounds by Mr Ryoichi Sasakawa, the millionaire Japanese industrialist and philanthropist who also helped to finance the WHO campaign which has eliminated smallpox. The transformation has been beautifully done and this charming little centre is now waiting to be used. It will seat 60 and projection apparatus is available. There is also a small 'Tutorial Room' seating 6–8, and on the ground floor a 'lounge' where a buffet meal can be arranged. It is adjacent to the Jenner Museum and there is fairly ample car parking. Nearby Berkeley Castle is an additional attraction. Anyone interested should apply to The Custodian, Jenner Museum, The Chantry, Berkeley.

C. Bruce Perry

